# Adsorption of heavy metal with modified eggshell membrane and the *in situ* synthesis of Cu–Ag/modified eggshell membrane composites

**DOI:** 10.1098/rsos.180532

**Published:** 2018-09-19

**Authors:** Yaqing Xin, Caihong Li, Jianing Liu, Jinrong Liu, Yuchen Liu, Weiyan He, Yanfang Gao

**Affiliations:** 1 College of Chemical Engineering, Inner Mongolia University of Technology, Hohhot 010051, People's Republic of China; 2 Zhongtian Synergetic Energy Co. Ltd., Ordos 017317, People's Republic of China

**Keywords:** biosorption, heavy metals, resource transformation, composites, 4-nitrophenol

## Abstract

The objectives of this study were to remove heavy metals from wastewater through the biosorption method with modified biomass as an effective sorbent and to prepare metal/biomass composites with the same modified biomass as a direct template. Eggshell membrane (ESM) was selected and modified to adsorb heavy metals. Adsorption of metal ions on the modified ESM (MESM) might be attributed to electrostatic interaction, ion exchange and coordination effect with chelating ligands containing N and S on the surface of the MESM. The pH of the solution was a key factor affecting the adsorption. The Cu–Ag/MESM composites with uniform Cu–Ag NPs were prepared with MESM as matrices, and with Cu^2+^ and Ag^+^ adsorbed as metal sources. The Cu–Ag/MESM showed excellent catalytic performance in the reduction of 4-nitrophenol to 4-aminophenol in the aqueous phase. Because of the high stability of the Cu–Ag NPs supported on the macro-dimension supporter, Cu–Ag/MESM can be easily separated after the catalytic reaction and recycled.

## 1. Introduction

Wastewater containing heavy metals from industries, such as tanneries, mining operations, fertilizers, batteries, chemical manufacturers and pesticides, has been a major pollutant in the environment [1,2]. Owing to their non-biodegradability and persistence, heavy metals tend to accumulate and enter the food chains causing numerous diseases and disorders in humans [3]. Therefore, efficient removal of heavy metal ions from wastewater is of extreme importance.

Up to now, methods to remove heavy metal ions from aqueous solution consist mainly of chemical precipitation, ion exchange, adsorption, membrane filtration, coagulation and flocculation [4–6]. Although there are significant disadvantages in these methods, such as incomplete removal, high-energy requirements and the production of secondary pollution [7], the adsorption method is more suitable for the adsorption of heavy metals in low concentration because it is easy to operate under field conditions, with renewable adsorbents, high recovery rate of heavy metals and zero sludge production [8–10]. However, the widely used adsorbent-active carbon is expensive with a short service life and high cost of regeneration, which limits the application of activated carbon adsorption to a certain extent. Most importantly, in the process of adsorbent regeneration, it may cause waste of resources and secondary pollution of the heavy metals. Therefore, during the adsorption of heavy metals from wastewater, it is necessary to prepare cheap and efficient adsorbent materials and to study the method of desorption and recycling of heavy metals after adsorption. Because of the low cost, high efficiency and biodegradability of the adsorbents, biosorption has recently been paid much attention in the removal of heavy metals [11]. Reported results showed that many biomass materials had the potential to adsorb heavy metals [12–16]. Typically, adsorption ability of natural biomass materials is limited; thus, physical and chemical modification are required to improve the adsorption capacity [2,17], of which chemical modification is more commonly used because of its simplicity and efficiency. Acids, alkalis, minerals, organic acids, and oxidants are often used as modifiers in order to increase bonding sites and eliminate surface interference of biomass in chemical modification. In addition, ionic polymers containing more functional groups which can bond with metal ions can be used to encapsulate the biomass to increase its adsorption capacity. Polyethylenimine (PEI)-functionalized eggshell membrane (PEI-ESM) was prepared through the cross-linking reaction between aldehydes in glutaraldehyde and functional groups such as amines and amides in ESM. Its maximum equilibrium adsorption capacity towards Cr(VI) can reach about 160 mg g^−1^ with an initial pH of 3.0 [18]. Thiol-functionalized ESM was synthesized from ESM through thiol functionalization based on the reduction of disulfide bonds in ESM with ammonium thioglycolate. With thiol-functionalized ESM as an adsorbent, the removal of Cr(VI), Hg(II), Cu(II), Pb(II), Cd(II) and Ag(I) from aqueous water was investigated. The experimental results revealed that the adsorption abilities of the thiol-functionalized ESM towards Cr(VI), Hg(II), Cu(II), Pb(II), Cd(II) and Ag(I) were improved by 1.6-, 5.5-, 7.7-, 12.4-, 12.7- and 21.1-fold, respectively, compared to those of the ESM itself [19]. On the other hand, some studies also indicated that biomass materials displayed an astonishing variety of sophisticated nanostructures that were difficult to obtain even with the most technologically advanced synthetic methodologies [20], which suggested that such materials could be used as novel platforms to immobilize and synthesize materials with specific structures.

It is well known that a smaller size of metal nanoparticles implies a larger percentage of metal atoms exposed at surface. With the decrease in particle size, however, the metal particles are very easy to aggregate to minimize their surface energy, resulting in poor stability, coupled with a significant reduction in the separation and recycling of the practical applications [21,22]. Therefore, it is very important to synthesize metal nanomaterials with well dispersion, excellent stability and easy recovery.

ESM is composed of a double layer membrane structure and a protein-based interwoven fibrous structure containing glycoproteins such as collagen (types I, V and X) and amino acids (glycine alanine and uronic acid) [23,24]. Multifield applications of ESM, such as enzyme immobilization [25], metal ion removal [19], electrode materials of super capacitor [26], solid-phase extraction biosorbent [27,28] and metal/semiconductor composites [22,29], have started to attract wide attention in recent years. Studies also showed that surface modified ESM (MESM) could act as an efficient supporter to prepare stable Ag [30], Pd and Pt nanoparticles-MESM composites [31]. To the best of our knowledge, however, studies on adsorption of metal ions with ESM as sorbent, and the *in situ* preparation of metal/biomass composites with ESM as the template, have not been reported.

In this study, we described the removal of heavy metals from aqueous solution with MESM as a sorbent and the *in situ* preparation of metal/MESM composites with MESM as a direct template. During the adsorption, metal ions were adsorbed due to the complicated electrostatic cooperating effects and the bonding interactions to construct a stable linkage between MESM fibre and metal ions, thus forming metal ion/MESM composites, and then the coordinated metal ions on the MESM could be reduced with NaBH_4_. The reduction of 4-nitrophenol (4-NP) was chosen as a model reaction to investigate catalytic behaviours of the prepared metal/MESM composites. Physical and chemical properties of as-prepared samples were also characterized to explore the mechanism of adsorption and the formation of metal/MESM. Results showed that metal nanoparticles were obtained on the macro-dimension support MESM without aggregation, and metal/MESM composites could be easily separated from the reaction mixture without nanoparticles losing significantly. Meanwhile, because metal nanoparticles were loaded on the protein framework of MESM, the stability of supported nanoparticles was improved. This study will provide an economical and environment-friendly new approach to the removal of heavy metals from wastewater and their use. The simulated wastewater in this work was collected from the east Wu Qi mining area. According to the characteristics of local mineral resources, the reserves of Zn, Cu, Ag and Cd were large; therefore, Cu and Ag were chosen as models to study the effect of representative heavy metal ions during the adsorption and preparation of functional materials.

## 2. Material and methods

### 2.1. Chemicals and reagents

All chemicals were of analytical grade and used directly without further purification. Sodium sulfide (Na_2_S), sodium hydroxide (NaOH), sodium borohydride (NaBH_4_) and 4-NP (99%) were purchased from Tianjin Chemical Reagents Co. (Tianjin, China). Stock solutions of heavy metal ions (1000 mg l^−1^) were prepared from Cu(NO_3_)_2_·3H_2_O and AgNO_3_ provided by Sinopharm Chemical Reagent Co., Ltd. (Shanghai, China). Standard solutions of copper, silver (100 µg ml^−1^) for the atomic absorption spectrophotometer were from Beijing National Institute of Standard Material P.R. China. Deionized water was used for all the experiments. The pH of the solution was adjusted using 0.1 M NaOH or 0.1 M HNO_3_ solution.

### 2.2. Preparation process of MESM

Eggshell collected from a school canteen was immersed in 0.05 M acetic acid solution for 1 h, so that the ESM could be peeled off easily by hand. Then the ESM was washed with deionized water to remove the residual impurities and dried in vacuum at 35°C for future use. One gram of ESM was suspended in 400 ml of NaOH (0.1 M) solution at 35°C for 4 h, and then the ESM was taken out and rinsed with deionized water three times. NaOH-modified ESM was immersed in 400 ml of Na_2_S (0.2 M) solution at 35°C for 12 h, and then the MESM was washed repeatedly with deionized water and dried at 35°C. Finally, the obtained MESM was cut into small pieces (about 5 × 8 mm) for the adsorption test.

### 2.3. Adsorption experiments

To investigate the effect of pH on the adsorption capacity, 0.1 g of MESM was added into 100 ml of the target metal ions solution (Cu^2+^, Ag^+^ and Cu^2+^, Ag^+^ mixed solution) under different pH conditions. The initial concentrations of Cu^2+^ and Ag^+^ were both 100 mg l^−1^. The flasks were shaken at 150 r.p.m. and 35°C for 12 h. The uptake capacity of MESM for heavy metals was determined by analysing the metal concentrations of the solution before and after adsorption. The adsorption capacity of the adsorbent was calculated according to the equation as follows:

2.1 $$q_{t}=\frac{(C_{0}-C_{t})V}{m},$$

where *C*_0_ (mg l^−1^) and *C_t_* (mg l^−1^) are the initial and time *t* concentration of the metal ions in the solution, respectively; *m* (g) is the mass of the adsorbent; *V* (ml) is the volume of the solution. All of the results were performed three times under the same conditions and the data were recorded as an average value.

### 2.4. Preparation process of Cu–Ag/MESM composites

Controlling the adsorption capacity for Cu and Ag to 20 mg g^−1^ for each, the Cu^2+^–Ag^+^/MESM was prepared. For comparison, Cu^2+^/MESM and Ag^+^/MESM intermediates were prepared by controlling the adsorption capacity for single metal ion at 40 mg g^−1^ and under the same other conditions, and the above intermediates were thoroughly washed with deionized water. In order to obtain the Cu/MESM, Ag/MESM and Cu–Ag/MESM composites, the MESM-loaded metal ions were transferred into 10 ml deionized water, and 10 ml NaBH_4_ (0.1 M) was introduced drop by drop to convert the ions into metallic element nanoparticles under magnetic stirring and ice bath. Then prepared samples were thoroughly rinsed with deionized water and dried in vacuum for further characterization and catalytic application.

### 2.5. Catalytic reduction of 4-nitrophenol

The catalytic reduction of 4-NP by NaBH_4_ was chosen as a model to investigate the catalytic performance and reusability of the Cu–Ag/MESM composites. In a typical experiment, 60 mg of prepared composites were added into a mixed solution containing 25 ml of deionized water and 1 ml of 4-NP (2 mM). N_2_ was then purged through the solution for 10 min to remove the dissolved O_2_. Then, 4 ml of fresh prepared NaBH_4_ solution (0.33 M) was added under continuous stirring at room temperature. The conversion of 4-NP was tested by the characteristic absorption peak of 4-NP at 400 nm with UV–vis absorption spectra. For comparison, Cu/MESM and Ag/MESM were also used as catalysts for the reduction of 4-NP under the same conditions. The reusability of the Cu–Ag/MESM composites as a catalyst was investigated via the catalyst recycling experiments.

### 2.6. Characterization methods

Fourier transform infrared (FTIR) spectra of ESM, MESM, Cu^2+^/MESM and Ag^+^/MESM were obtained with the Nicolet Nexus 670 in the range of 400–4000 cm^−1^. Surface morphologies of natural ESM, MESM and Cu–Ag/MESM composites were measured using a scanning electron microscope (SEM, Hitachi S-4800) equipped with energy-dispersive spectroscopy (EDS). Crystal structure and composition of the samples were characterized by X-ray diffraction (XRD) using a German Bruker D8 X-ray diffractometer and operated with Cu Ka radiation (*λ* = 1.5418 Å). X-ray photoelectron spectra (XPS) were monitored using an ESCALAB 250XI spectrometer, Thermo-Fisher Ltd.

## 3. Results and discussion

### 3.1. Characterization of the biosorbent

**Figure 1.**
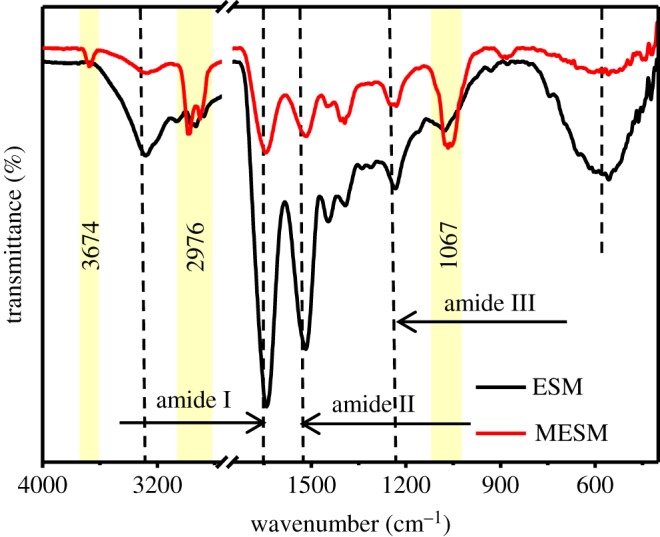
FTIR spectra of ESM and MESM.

**Figure 2.**
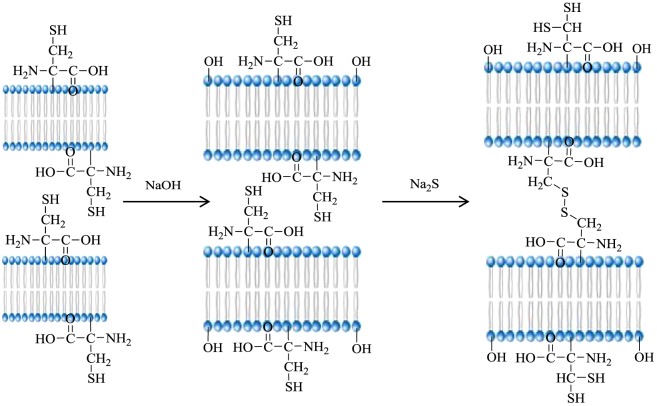
Schematic for modification mechanism of ESM.

The FTIR spectra of ESM and MESM are shown in figure 1. Both samples had absorption peaks at 1635, 1527 and 1240 cm^−1^, corresponding to the amide I (−COO^−^ stretching vibration), amide II (N−H in plane bending vibration coupled with C−N stretching vibration) and amide III bands of the proteins in ESM, respectively [32], indicating that the structure of the ESM framework had been kept in the MESM. Compared with the spectra of the natural ESM, several new peaks appear in the spectra of the MESM. The peak at 3674 cm^−1^ corresponded to the presence of free −OH groups which did not take part in hydrogen bonding [33]. The peak at 2976 cm^−1^ was attributed to the symmetric and asymmetric C–H stretching vibration of methyl, methylene and methoxy groups [34]. And an obvious absorption band at 2974 cm^−1^ corresponded to the characteristic peak of –SH, indicating that the sulfur element was introduced successfully into the ESM skeleton after the treatment with sodium sulfide. The peak at 1067 cm^−1^ reflected the form of S-S bond. Because ESM contained not only the general elements (C, H, O, N) but also sulfur from cysteine which formed an SH bond in the keratin of ESM, when cysteine was heated or was subjected to chemical reagent, it underwent a chemical reaction to form a disulfide bond (S–S) [35]. The clear shift of the weak broad stretching peak at 609 cm^−1^ corresponded to C-S [36] because HS^−^ from hydrolysis of Na_2_S replaced H in –CH_2_–SH of cysteine. Therefore, it could be assumed that the modification mechanism of ESM could be shown in figure 2.

### 3.2. Effect of pH on the adsorption and adsorption mechanism of MESM

The pH value of solution played a significant role in affecting the adsorption [37]. With pH < 2, functional groups on the surface of MESM were protonated to form the positively charged sites and electrostatic repulsion occurred between metal ions and the positive surface of the MESM, leading to the decrease in the level of metal ion adsorption on MESM due to the electrostatic interaction. Meanwhile, the concentration of H^+^ was high at a low pH, thus the hydroxyl group on the surface of MESM was neutralized by H^+^ and favoured the adsorption of this species leading to its competition with metal ions and the decrease of metal ion adsorption. Moreover, the precipitation of Cu(OH)_2_ and AgOH took place at pH > 6.0 [38,39]. Therefore, the pH range of the adsorption solution was from 2.0 to 6.0.

**Figure 3.**
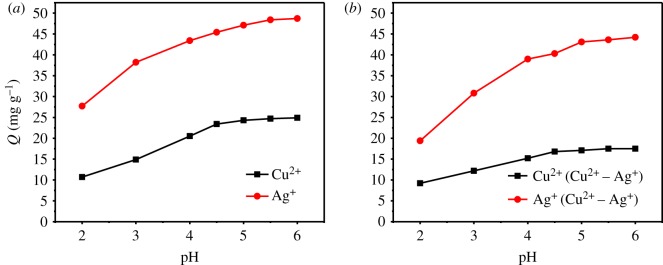
Effect of pH on adsorption of Cu^2+^ and Ag^+^ by MESM for single solution (*a*) and binary mixed solution (*b*).

Effects of pH on adsorption of MESM towards Cu^2+^ and Ag^+^ are shown in figure 3. In both single and mixed solutions, pH slightly influenced the adsorption capacity of MESM towards Cu^2+^, and the Cu^2+^ uptake on the MESM was increased with the increase of solution pH from 2 to 4.5 and reached a maximum adsorption capacity at pH = 4.5. Then, the adsorption capacity remained unchanged until pH = 6. Ag^+^ adsorption capacity was affected significantly by the solution pH value. At a low pH around 2.0, the Ag^+^ adsorption capacity was relatively low. And with the increase of pH, adsorption capacity was increased significantly and reached a maximum adsorption capacity at pH = 5.5.

Compared with the adsorption of metal ions in single solution (figure 3*a*), the adsorption of each kind of metal was affected by the presence of other metal ions in the mixed solution (figure 3*b*). The presence of Ag^+^ led to the decrease of adsorption capacity of Cu^2+^, and the adsorption capacity was decreased from 24 mg g^−1^ in single solution to 15 mg g^−1^ in binary mixed solution. The adsorption capacity of Ag^+^ was decreased slightly in the solution with the presence of Cu^2+^. Moreover, compared with single solution, Ag^+^ adsorption was significantly affected by the pH of solution in binary mixed solution. At a low pH around 2.0, the Ag^+^ adsorption capacity was only about 18 mg g^−1^, and with the increase of pH, adsorption capacity was about 49 mg g^−1^ at pH = 5.0–6.0.

**Figure 4.**
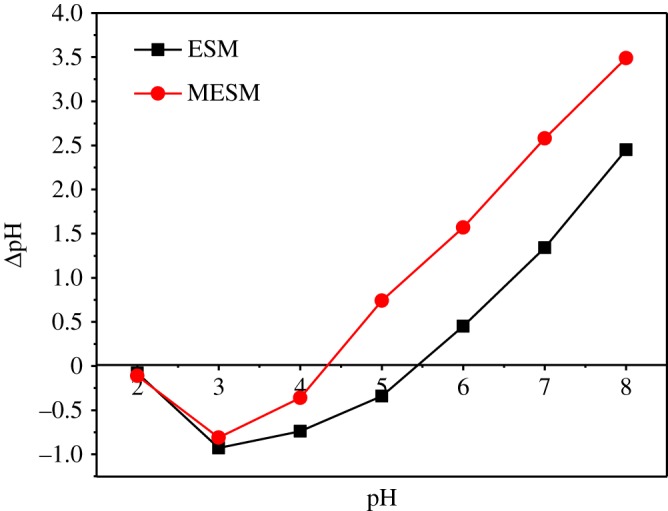
pH_pzc_ of ESM and MESM.

It is well known that pH at zero point charge (pHzpc) is an important parameter, which determines the surface behaviour of the adsorbent [5]. At pH below pHzpc of the adsorbent, the surface is positively charged, and above pHzpc, the adsorbent surface is negatively charged. Figure 4 shows the results of the pHpzc of ESM and MESM and pHzpc values of the ESM and MESM are 5.51 and 4.5, respectively. When pH < 4.5, because MESM formed the positively charged sites, electrostatic repulsion occurred between metal ions and the positive surface of the MESM, leading to the decrease of metal ion adsorption on MESM. When pH > 4.5, MESM formed the negatively charged sites, and electrostatic attraction occurred between metal cations and negatively charged sites on the surface of MESM. This result indicated that the electrostatic interaction was involved in the adsorption process.

Furthermore, solution pH change with different initial concentration before and after adsorption was further tested to analyse the interaction between metal ions and adsorbent. Solution pH values before (pH_0_) and after (pH_f_) adsorption with different initial concentrations are listed in table 1. The results showed that pH_f_ was lower than pH_0_, which indicated an increase in H^+^ concentration in the solution, implying that adsorption of metal ions on MESM was mainly through ion exchange. In ion exchange process, H^+^ would be released from the functional groups, such as –SH, –OH and –COOH on the surface of MESM. Meanwhile, metal ions were adsorbed on the active sites of the adsorbent to replace and release H^+^, and the possible reactions were shown as follows:

3.1 $$\text{nR}-\text{OH}+{\text{M}}^{n+}={\left(\text{R}-\text{O}\right)}_{n}^{-}\;{\text{M}}^{n+}+\text{n}{\text{H}}^{+}$$

3.2 $$\text{nR}-\text{COOH}+{\text{M}}^{n+}={\left(\text{R}-\text{COO}\right)}_{n}^{-}\;{\text{M}}^{n+}+\text{n}{\text{H}}^{+}$$

3.3 $$\;\text{and}\;\text{nR}-\text{SH}+{\text{M}}^{n+}={\left(\text{R}-\text{S}\right)}_{n}^{-}\;{\text{M}}^{n+}+\text{n}{\text{H}}^{+}.$$

**Table 1.** pH values before and after adsorption for different initial solution concentration.

	Cu^2+^		Ag^+^		Cu^2+^–Ag^+^	
*C*_0_ (mg g^−1^)	pH_0_	pH_f_	pH_0_	pH_f_	pH_0_	pH_f_
20	5.5	4.85	5.5	4.55	5.5	5.12
50	5.5	4.77	5.5	4.30	5.5	5.03
75	5.5	4.93	5.5	4.23	5.5	4.92
100	5.5	5.04	5.5	4.25	5.5	4.32

**Figure 5.**
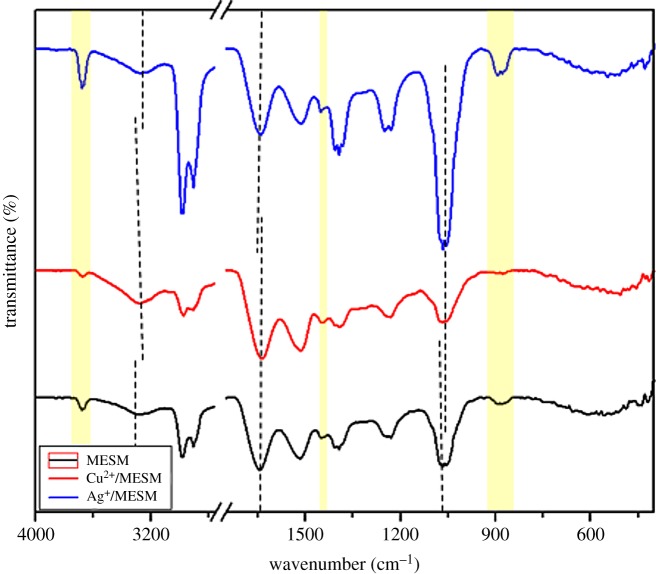
FTIR spectra of MESM, Cu^2+^/MESM and Ag^+^/MESM composites.

In order to further demonstrate the adsorption mechanism of MESM to Cu^2+^ and Ag^+^, the infrared spectra of MESM after the loading of Cu^2+^ and Ag^+^ were analysed. As shown in figure 5, the infrared spectra of the main functional groups of Cu^2+^/MESM and Ag^+^/MESM had changed compared with those of MESM. The hydroxyl peaks of 3282 cm^−1^ moved to 3279–3275 cm^−1^, C–OH bond at 1080 cm^−1^ had a small shift, the asymmetric and symmetrical stretching vibration peaks of C=O at 1639 and 1449 cm^−1^ moved slightly, the peak near the sulfhydryl groups at 2974 cm^−1^ was weaker and new peaks appeared from 895 to 905 cm^−1^, which indicated that the hydroxyl, carboxyl and sulfhydryl groups on MESM were involved in the adsorption process of Cu^2+^ and Ag^+^. According to the effect of pH on the adsorption process, the adsorption process of MESM towards Cu^2+^ and Ag^+^ might mainly include electrostatic interaction between negatively charged sites on the surface of MESM and metal cations, the exchange of ions with H^+^ in the hydroxyl, carboxyl, sulfhydryl groups and the coordination effect with chelating ligands containing N and S atoms of MESM [40].

### 3.3. Fabrication and characterization of Cu–Ag/MESM composites

**Figure 6.**
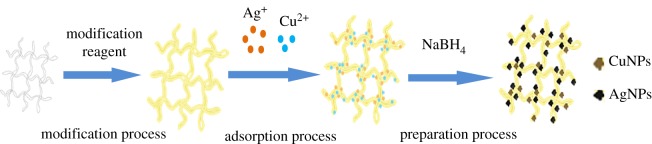
Schematic of the formation procedure of Cu–AgNPs with MESM as biotemplate.

On the basis of the adsorption results of heavy metal on MESM, Cu–Ag/MESM composites were prepared *in situ* with MESM as matrices and with Cu^2+^, Ag^+^ adsorbed as metal sources, and the fabrication procedure of the Cu–Ag/MESM composites is illustrated in figure 6. Theoretically, Cu^2+^ and Ag^+^ can be uniformly anchored and distributed on MESM fibres after the adsorption process with the help of the interactions between the functional groups (–SH, –OH and –COOH) on the surface of fibres and the adsorbed metal ions. In addition, the interconnected porous structure of MESM could provide good permeability to allow the Cu^2+^ and Ag^+^ to infiltrate into the inner fibres of MESM. With the introduction of NaBH_4_, the chelated Cu^2+^ and Ag^+^ on the surface of MESM can be reduced into Cu^0^ and Ag^0^ elementary substance. The synthesized Cu–Ag/MESM was subsequently stabilized due to the interaction between the Cu–AgNPs and the surface active sites of MESM.

**Figure 7.**
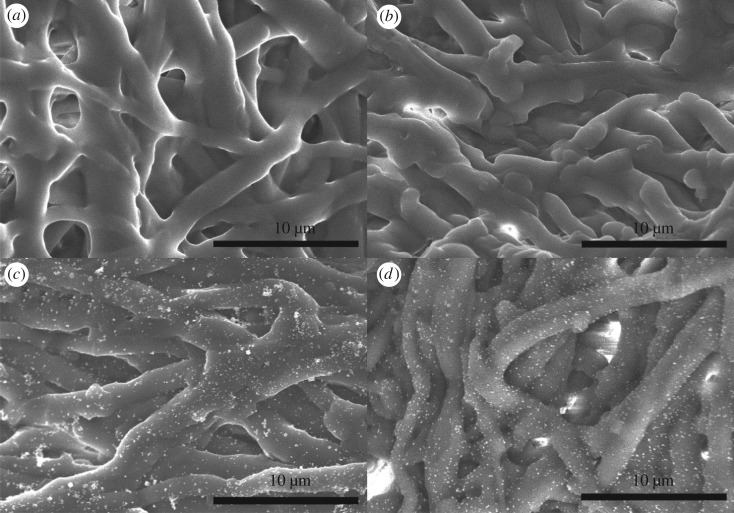
SEM images of natural ESM (*a*), MESM (*b*), Cu^2+^–Ag^+^ /MESM (*c*) and Cu–Ag/MESM (*d*).

The morphologies of the samples were characterized with SEM. As shown in figure 7*a*, natural ESM exhibited a three-dimensional network structure that was composed of interwoven and coalescing protein fibres, and the diameter of protein fibres was about 0.5–1 µm. High surface area caused by the interwoven structure was beneficial to the immobilization of nanoparticles. Meanwhile, this macroporous structure could endow ESM with good permeabilities that allowed reactants to contact the inner fibres sufficiently. After ESM was modified (figure 7*b*), MESM almost retained the macroporous network structure of ESM. However, a rougher surface was observed, which indicated that the modified reagent acted on the fibre surface of ESM. Figure 7*c* shows the image of Cu^2+^–Ag^+^/MESM. The MESM fibres are almost ‘coated’ with particles of metal salt formed from metal ions (Cu^2+^ and Ag^+^). Figure 7*d* shows the image of Cu–Ag/MESM prepared. The interconnected fibrous structure of ESM was also well-retained, and the surface of fibres was decorated by many well-dispersed small-sized nanoparticles with uniform spherical shape. And these spheroids composed of Cu–Ag nanoparticles were bound so closely to the MESM fibres as to achieve Cu–Ag/MESM hybrid composites. The structure and composition of the Cu–AgNPs/MESM composites were further characterized with EDS, XRD and XPS.

**Figure 8.**
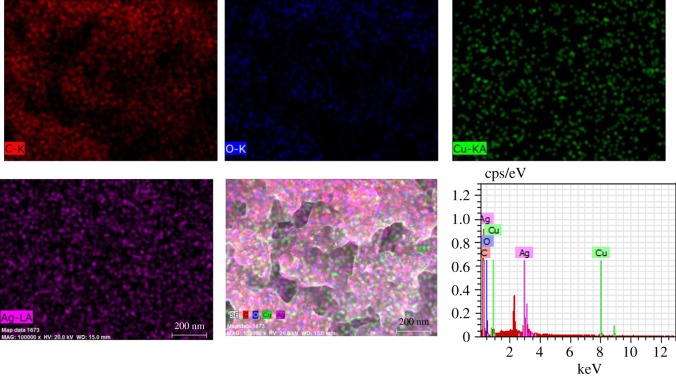
SEM-EDS elemental mapping image of Cu–Ag/MESM composites.

As shown in figure 8, EDS spectrum analysis of the Cu–Ag/MESM composites confirmed that Cu and Ag nanoparticles were distributed on the intricate lattice network of MESM and revealed that Cu and Ag nanoparticles on the protein framework consisted of C and O.

**Figure 9.**
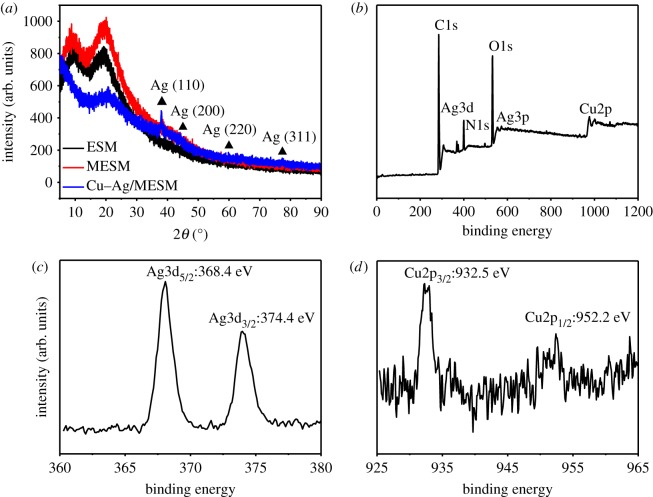
XRD patterns of Cu–Ag/MESM composites (*a*) and XPS fully scanned spectra of Cu–Ag/MESM composites (*b*), detailed spectra of the Ag 3d (*c*) and Cu 2p (*d*) binding energies.

The XRD patterns of the natural ESM, MESM and as-prepared Cu–Ag/MESM composites are shown in figure 9*a*. ESM and MESM exhibited broad diffraction peaks at around 10° and 20.6°, which could be attributed to the ESM protein fibres containing plenty of amines, amides and carboxylic groups [41]. The Cu–Ag/MESM composites exhibited four diffraction peaks at 38.0°, 44.1°, 64.3° and 77.5°, which was consistent with the diffraction from the (111), (200), (220) and (311) planes of the face-centred cubic Ag (JCPDS NO. 04–0783), respectively. However, characteristic diffraction peaks of CuNPs had not been detected from the patterns of the composites, suggesting the formation of very small-sized nanoparticles, which had been observed in many metal/semiconductor nanoparticles immobilized on organic framework [42].

In order to further characterize the chemical state of copper and silver in Cu–Ag/MESM composites, XPS of the samples were investigated. As shown in figure 9*b* with the survey scan range of 0–1200 eV, C1s, N1s, O1s, Ag3p, Ag3d and Cu2p could be observed in Cu–Ag/MESM over. The peaks located at 286.2 and 532.8 eV corresponded to the C 1s and O 1s binding energies, respectively. The high percentage of elemental O might come from the rich oxygen-containing functional groups on the Cu–Ag/MESM surface. The peaks of the Ag 3d and Cu 2p binding energy were also detected. Magnified Ag 3d peaks are shown in figure 9*c*. Two peaks at 368.4 and 374.4 eV corresponded to Ag 3d_5/2_ and Ag 3d_3/2_ binding energies, respectively. A similar phenomenon could be observed for Cu 2p (figure 9*d*). In figure 9*d*, the binding energies at 932.5 and 952.2 eV corresponded to Cu 2p_3/2_ and Cu 2p_1/2_, respectively. These results confirmed that the surface of composite samples contained metallic AgNPs (Ag^0^) and CuNPs (Cu^0^).

### 3.4. Catalytic properties of Cu–Ag/MESM

The reduction of 4-NP to 4-aminophenol (4-AP) in the presence of NaBH_4_ was chosen as a model reaction to evaluate the catalytic activity of the Cu–Ag/MESM prepared. 4-NP generated from pesticide and herbicide can cause water pollution, while its reduced product (4-AP) is an important intermediate for the manufacture of antipyretic and analgesic drugs [43]. Although this reaction is thermodynamically favourable (E_0_|4-NP/4-AP = −0.76 V and ${\text{H}}_{3}\text{B}{\text{O}}_{3}/BH_{4}^{-}=-1.33\;\text{V}$ versus normal hydrogen electrode), due to the kinetic barrier caused by the large potential difference between donor and acceptor molecules, it does not proceed without catalysts [44]. In this study, when metal nanoparticles are used as catalysts, they can act as electronic relay systems to transfer electrons from the donor $\text{B}{\text{H}}_{4}^{-}$ to the acceptor nitro group of 4-NP [45].

**Figure 10.**
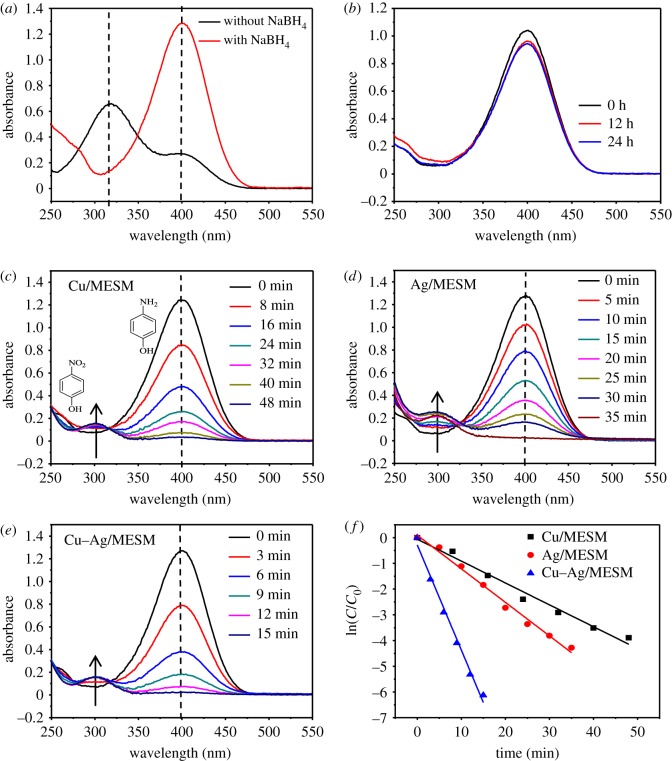
UV–vis adsorption spectra of 4-NP solution with and without NaBH_4_ (*a*). Time-dependent UV–Vis spectra monitoring the catalytic reduction of 4-NP with NaBH_4_: in the presence of MESM (*b*), Cu/MESM (*c*), Ag/MESM (*d*) and Cu–Ag/MESM (*e*). Plots of ln (*C_t_*/*C*_0_) corresponding to the reaction time for the reduction of 4-NP catalysed by Cu/MESM, Ag/MESM and Cu–Ag/MESM, respectively (*f*).

In all experiments, the initial concentrations of 4-NP and NaBH_4_ were kept at 2 mmol l^−1^ and 0.33 mol l^−1^, respectively. Figure 10 presents the typical time-dependent UV-vis absorption spectra during the reaction. In figure 10*a*, the single 4-NP solution showed an absorbance peak of 317 nm, which shifted from 317 nm to 400 nm after the addition of NaBH_4_ due to the formation of 4-nitrophenolate ions via deprotonation. In figure 10*b*, the UV–vis spectra of adsorption peak at 400 nm did not change even for one day when only MESM was added into the reaction system, which suggested that MESM itself had no catalytic activity except for being used as a catalyst carrier. In figure 10*c*–*e*, the absorption of 4-NP ions at 400 nm started to decrease. Meanwhile, a new UV-vis peak at 300 nm appeared due to the generation of the reduction product 4-AP in the presence of Cu/MESM, Ag/MESM and Cu–Ag/MESM, respectively. And the bright yellow colour of 4-NP solution disappeared completely within a certain period of time, indicating that the 4-NP was completely converted to 4-AP, which confirmed the catalytic roles of immobilized CuNPs and AgNPs on the reduction reaction. In order to evaluate the catalytic performance of the three composites as prepared, the reduction rates of 4-NP were calculated. The concentration of NaBH_4_ was significantly excessive compared with that of 4-NP. Thus, the concentration of NaBH_4_ could be considered as a constant, and the reduction rate could be assumed to be independent of the concentration of NaBH_4_. Therefore, the pseudo-first-order rate kinetics could be applied to evaluate the catalytic reaction rate of 4-NP with metal/MESM composite as a catalyst [46]. The equation is described as follows:

3.4 $$r_{t}=\frac{-\text{d}C_{t}}{\text{d}t}=kC_{t},$$

where *r_t_* was the consumption rate of 4-NP at time *t*, *C_t_* was the concentration of 4-NP at time *t* and *k* was the first-order rate constant.

*k* was determined from a linear plot of ln(*C_t_*/*C*_0_) versus reaction time *t* of the reduction of 4-NP. *C_t_*/*C*_0_ was monitored from the respective absorbance intensity of 4-NP. The concentration was proportional to its absorbance intensity in the medium. Figure 10*f* shows a good linear correlation of ln(*C_t_*/*C*_0_) versus time (*t*), confirming the pseudo-first-order kinetics, and the rate constants (*k*) of the catalytic reaction with Cu/MESM, Ag/MESM and Cu–Ag/MESM as catalysts were estimated to be 8.55 × 10^−2^, 13.50 × 10^−2^ and 40.21 × 10^−2^ min^−1^, respectively, which suggested that the catalytic properties of the composites were Cu–Ag/MESM > Ag/MESM > Cu/MESM. This trend can be attributed to the formation of the bimetallic nanoparticles, which can change the electronic structure, in particular the d-band centre of the involved metal atoms highly relevant to catalytic activity. Moreover, the electron density on the surface of the Cu–AgNPs was higher than that of the monometallic nanoparticles because electron transfer from Cu to Ag would cause a synergistic electronic effect [47].

Recycling performance is another key factor to evaluate practical application of the catalyst. The reusability and stability of the catalyst were assessed through cycling and reuse of the Cu–Ag/MESM, and the composites were reused to catalyse the reduction reaction of 4-NP under the same reaction conditions. After the reaction, the composites were separated from the reaction mixture through centrifugation, washed with water several times, dried in the vacuum oven and reused directly for the next run without additional supplementation of catalyst to the initial amount.

**Figure 11.**
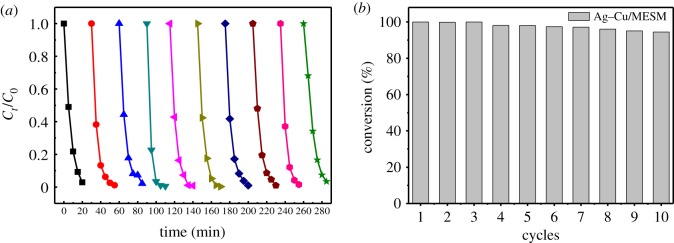
Recycling and reuse of Cu–Ag/MESM composite for the reduction of 4-NP to 4-AP (*a*). Conversion efficiency of 4-NP over 10 successive cycles (*b*).

The recycling results are shown in figure 11. The conversion rate of 4-NP can be retained at 94% after 10 cycles, and the slightly decrease of conversion rate might be partly caused by the inevitable loss of the amount of the catalyst during the washing and centrifugation. These results indicated that metal nanoparticles could be separated easily from the mixture without too much loss because they were immobilized on the macro-dimension support with a strong bind force.

## 4. Conclusion

ESM was modified to remove Cu^2+^ and Ag^+^ from aqueous solution, and Cu–Ag/MESM composites were prepared *in situ* with the MESM as matrices, and with adsorbed Cu^2+^ and Ag^+^ as metal sources. Results showed that the pH of the solution was a key factor for adsorption with MESM as an adsorbent. The Cu–Ag/MESM composites had shown an excellent catalytic performance in the reduction of 4-NP and excellent reusability. Therefore, this study will provide a facile, cost-effective, environmentally-friendly new method to apply biosorption to remove heavy metals from wastewater, and use heavy metal resources at the same time.

## Data accessibility

Data available from the Dryad Digital Repository: http://dx.doi.org/10.5061/dryad.4gp7g3g.
